# O.6.3-5 A national audit into the different levels of typical school provision of physical education, physical activity and sports in the Republic of Ireland

**DOI:** 10.1093/eurpub/ckad133.285

**Published:** 2023-09-11

**Authors:** Padraic Rocliffe, Brendan O'Keeffe, Ian Sherwin, Patricia Mannix-McNamara, Ciaran MacDonncha

**Affiliations:** University of Limerick, Ireland; padraic.rocliffe@ul.ie; University of Limerick, Ireland; padraic.rocliffe@ul.ie; University of Limerick, Ireland; padraic.rocliffe@ul.ie; University of Limerick, Ireland; padraic.rocliffe@ul.ie; University of Limerick, Ireland; padraic.rocliffe@ul.ie

## Abstract

International guidelines recommend sixty-minutes of moderate-to-vigorous-intensity physical activity (PA) daily for adolescents (WHO, 2020). Prevalence of physical inactivity is high and is regarded as a leading risk factor for death worldwide, contributing to the onset of non-communicable diseases with just 20% of adolescents meeting the recommended PA guidelines (Guthold et al., 2020). Physical inactivity cost $67.5 billion worldwide in 2013 and is estimated to reach over €110 billion in 2030 (WHO, 2022). Despite the worldwide adoption of school PE, PA and sport policies to promote PA and health, paralleled with significant investment, a gap in the literature exists that audits the different levels of typical school provision of PE, PA and sports.

Participating schools (n = 112) completed the validated PE, PA and sports provision evaluation index. A One-way ANOVA with Tukey Kramer's Post-Hoc test was performed to examine variation in the demographic profile relative to the indicators of provision. A proposed grade for each indicator of provision was established using a standardized, international grading system.

Half of schools had no outdoor all-weather surface (54.5%). Two thirds had no facilities to accommodate active transport (66.1%). One in five felt the percentage of the budget given towards PE, PA and sports was inadequate (19.6%). Regarding indicators of provision, partnerships received the lowest grade (C-) while facilities and equipment received the highest grade (B-). Provision scores for personnel were significantly higher for mixed schools (girls/boys) in comparison to girls schools. Provision scores for budget and partnerships were significantly higher for larger schools (>800) then small schools (<300).

The evidence suggests that while there has been a significant shift in focus both in policy development and the availability of financial resources to enhance typical school provision of PE, PA and sports for adolescent health, considerable modification of existing provision is required to potentiate positive impact.

